# Nonlinear Granger Causality between Health Care Expenditure and Economic Growth in the OECD and Major Developing Countries

**DOI:** 10.3390/ijerph15091953

**Published:** 2018-09-07

**Authors:** Liping Ye, Xinping Zhang

**Affiliations:** School of Medicine and Health Management, Tongji Medical College, Huazhong University of Science and Technology, Wuhan 430030, China; xiaoye3531@126.com

**Keywords:** health care expenditure, economic growth, Linear Granger causality test, Nonlinear Granger causality test

## Abstract

Differing from previous studies ignoring the nonlinear features, this study employs both the linear and nonlinear Granger causality tests to examine the complex causal relationship between health care expenditure and economic growth among 15 Organisation for Economic Co-operation and Development (OECD) and 5 major developing countries. Some interesting findings can be obtained as follows: (1) For Australia, Austria, and UK, linear and nonlinear Granger causality does not exist between them. A unidirectional linear or nonlinear causality running from economic growth to health care expenditure can be found for Ireland, Korea, Portugal, and India. For these seven countries, health or fiscal policy related to health spending will not have an impact on economic growth; (2) For Belgium, Norway, and Mexico, only a unidirectional linear causality runs from health care expenditure to economic growth, while bidirectional linear causality can be found for Canada, Finland, Iceland, New Zealand, Spain, Brazil, and South Africa. Especially for the US, China, and Japan, a unidirectional nonlinear causality exists from health spending to economic growth. To improve the quality of national health, life quality and happiness, these 13 countries should actively look to optimise policy related to health care expenditure, such as by enhancing the efficiency of health costs to promote sustainable economic development.

## 1. Introduction

The causal relationship between health care expenditure and economic growth is a widely concerned topic. In general, although health care expenditure is different depending on the country and time period, many countries regard health care expenditure as a means of promoting economic growth [1,2]. Increased spending on health care is able to provide people with the better care, which can improve the life expectancy, social welfare, and overall security of a society. Generally speaking, healthier workers can work more efficiently and for longer periods, indicating that spending more on health can improve people’s health conditions and result in raising labour productivity. Moreover, the improvement of labour efficiency can further promote the economy [3]. However, regarding the role of economic growth in health care expenditure, it can also increase the health spending. With the rapid economic development, the people will tend to pay more attention to life quality, so the demand for medical services also will generally increase year by year. Generally, countries with higher per capita income have more per capita health care expenditure, which also indicates that the rapid economic growth may contribute to an increase in the health spending [4].

Understanding the causality between health care expenditure and economic growth is important for health policy makers. If the health spending can indeed continue to promote the economic growth, then the policies that increase the health care expenditure can be used as an effective way to stimulate economic growth. On the contrary, if the health spending cannot effectively boost the sustainable economic growth, then the relevant health policies are not so important. Furthermore, because of the different development stages of one country, the economic structure will be different, and the utility of health care expenditure in accelerating economic development also must be approached from various angles. Therefore, the complex causal relationship between health care expenditure and economic growth is still unclear for countries in different stages of economic development. This is especially true for the developed and developing countries due to the differences in their economic systems, the scale of their economies, and their economic structures; here, the impact of the health care expenditure on the economic growth is also different, which requires further comparative analysis to reveal the complex causal relationship between them in different countries.

Due to the importance of the relationship between health care expenditure and economic growth, the literature in this field is increasing every year. Some scholars have estimated the income elasticity of health care expenditure to identify whether the health care expenditure was a necessary or luxury product. Blomqvist and Carter [5] found that the long-term income elasticity of health care expenditure was less than one, implying that health care could be regarded as necessary. Baltagi and Moscone [6] analysed the long-term economic connection between health costs and income in 20 Organisation for Economic Co-operation and Development (OECD) countries from 1971 to 2004, and found that health care was a necessity rather than a luxury. Rodríguez and Valdés [7] explored the long-term causality between Gross Domestic Product (GDP) and health care expenditure in Latin America, the Caribbean, and OECD countries from 1995 to 2014, and identified that health care expenditure was a necessity for Latin America and OECD countries. Abdullah et al. [8] analysed the characteristics of health care expenditure in 36 Asian countries from 1995 to 2013, and found that health care expenditure was necessary in these countries.

Some scholars also found that a bidirectional causality exists between health care expenditure and economic growth. Amiri and Ventelou [9] used a modified version of the Granger causality test proposed by Toda and Yamamoto to investigate the causal relationship between GDP and health care expenditure in OECD countries, and found that the bidirectional Granger causality could account for most countries. Amiri and Linden [10] studied 34 OECD countries from 1970 to 2012 and found that a bidirectional causality between GDP and health care expenditure existed in most countries. Halící-Tülüce et al. [11] implemented the dynamic panel model to analyse the relationship between health expenditure and economic growth in low- and high-income countries. Here, a reciprocal relationship in the short term was found. Using data spanning the period from 1995 to 2013, Chaabouni et al. [12] and Chaabouni and Saidi [13] found that a bidirectional causality existed between health expenditure and GDP growth in 51 countries (including low-, middle-, and high-income countries). However, other scholars found that the relationship between health care expenditure and economic growth was different because of the different samples used. Wang [14] explored the causality between health care expenditure and economic growth in 31 countries from 1986 to 2007, and the overall panel regression results showed that health care expenditure growth would stimulate economic growth. Aslan et al. [15] investigated seven industrialised countries from 1980 to 1999 and found that there was a bidirectional causality for France, Germany and the UK and a unidirectional linear causality running from health care expenditure to economic growth for Italy and Japan; however, there was no causal relationship for Canada and the US.

Regarding the relationship between health care expenditure and economic growth, the conclusions of the above studies are inconsistent, mainly due to the different methods or countries, different sample periods and so forth used. Even more remarkably, most of the previous studies have been limited to linear models. However, the shocks of significant economic events or the changes in economic policy regimes, which may result in mechanism transformation of the economic environment, would lead to structural changes in the pattern of economic development. Also, changes in health policies and significant fluctuations in the business cycle that directly impact government revenues may also potentially cause structural changes in health care expenditure patterns [16,17,18]. Therefore, it is not only necessary to analyse the linear relationship between health care expenditure and economic growth, but also to explore the nonlinear causality relationship between them.

From the growth trend of per capita health care expenditure in each country, there may be one or more structural breaks in the time series. Health care expenditure is often affected by changes in significant health or fiscal policies [19,20]; meanwhile, economic growth may also be affected by the shocks of a financial crisis or other significant economic events. These external impacts of policy changes and the financial crisis are likely to create structural changes in time series of health care expenditure and economic growth. Assuming structural breaks exist in the time series of health care expenditure and economic growth, then the nonlinear links need to be considered when their relationship is explored [21,22,23,24,25]. However, the linear Granger causality test method [26] that is based on the linear relationship between variables cannot capture the nonlinear causality. Diks and Panchenko [27] modified the nonlinear Granger causality test method proposed by Hiemstra and Jones [28], which could effectively address these limitations. The modified method has been widely used in many areas, such as macroeconomic and financial areas [29,30,31]. Furthermore, previous studies have rarely analysed non-OECD countries [32]. So to analyse the impact of the economic development phase on the causal relationship between health care expenditure and economic growth, in addition to the centralised analysis of OECD countries, this study also takes five major developing countries (China, Brazil, India, Mexico, and South Africa) as the research objects, hoping to provide more specific empirical results to further provide decision support for the relevant health management and policy makers. This study mainly has the following two contributions: (1) The nonlinear Granger causality between health care expenditure and economic growth comparing with the linear test is analysed in multiple countries; (2) A comparative analysis for developed and developing countries on the causality between health care expenditure and economic growth is explored.

## 2. Methods and Data Sources

### 2.1. Linear Granger Causality Test

The linear Granger causality test [26] is adapted to identify the linear relationship between health care expenditure and economic growth. First, the augmented Dickey-Fuller (ADF) unit root test [33] is used to explore the stationary characteristics of per capita health expenditure and per capita GDP time series. If all of the time series is stationary, the Vector autoregression (VAR) model proposed by Sims [34] can be used for the linear Granger causality test. In this study, the bivariate VAR model is constructed as follows:

(1) $$∆Y_{t}=a_{1}+\overset{m}{\underset{i=1}{\sum }}b_{1i}∆X_{t-1}+\overset{m}{\underset{j=1}{\sum }}c_{1j}∆Y_{t-1}+\varepsilon_{1t}$$

(2) $$\Delta X_{t}=a_{2}+\sum_{i=1}^{m}b_{2i}\Delta X_{t-i}+\sum_{j=1}^{m}c_{2j}\Delta Y_{t-j}+\varepsilon_{2t}$$

where $a_{1}$ and $a_{2}$ represent the intercept terms; b and c represent the estimated coefficients; and m represents the optimal lag order, which is determined by the Schwarz information criterion (SIC). Because the Akaike information criterion (AIC) would overfit the data and that the SIC is a better criterion for this application according to Koehler and Murphree [35]. The null hypothesis is that *X* cannot strictly Granger cause *Y* in Equation (1), and *Y* cannot strictly Granger cause *X* in Equation (2), which is represented by $b_{1i}=0$ and $c_{2j}=0$ (i=1,2,…,m; j=1,2,…,m).

### 2.2. BDS Test

It is necessary to test whether the health care expenditure and GDP time series have nonlinear characteristics when it comes to exploring their nonlinear Granger causality. In this study, the BDS test proposed by Brock et al. [36] is used to detect the nonlinearity of the time series, which is primarily based on the concept of a correlation integral [37]. The null hypothesis for the BDS test is that the data is independent and identically distributed. Given a m-dimensional time series, $X_{t}$, and its associated observations, $\left(X_{t},X_{t+1},\ldots ,X_{t+m-1}\right)$, the correlation integral is defined by Brock et al. [36] as follows:

(3) $$C_{m}(T,e)=\sum_{t=1}^{T_{m}-1}\sum_{s=t+1}^{T_{m}}I(X_{t}^{m}X_{s}^{m},e)\times \frac{2}{T_{m}(T_{m}-1)}$$

where $I(X_{t}^{m},X_{s}^{m},e)$ is an indicator function, and the form is as follows:

(4) $$I(X_{t}^{m},X_{s}^{m},e)=\left\{ \begin{array}{l} 1,\begin{array}{cc} \; & \left\|X_{t}^{m},X_{s}^{m}\right\|<e \end{array}\\ 0,\begin{array}{cc} \; & otherwise \end{array} \end{array}\right.$$

where $\left\|X_{t}^{m},X_{s}^{m}\right\|$ measures the Euclidian distance between $X_{t}^{m}$ and $X_{s}^{m}$. $T_{m}$ is the sample size, and T can be divided into subsamples, $T_{m}$, of m-dimension vectors. The correlation integral measures the segment of $\left(X_{t}^{m},X_{s}^{m}\right)$ within a range of maximum distance of e, so the BDS test statistic is defined as follows:

(5) $$W_{m}(T,e)=\frac{\sqrt{T}\left[C_{m}(T,e)-C_{1}{\left(T,e\right)}^{m}\right]}{\sigma_{m}(e)}$$

where T represents the sample size, and $\sigma_{m}(e)$ represents the standard deviation. The BDS test statistic, for example, $W_{m}(T,e)$ obeys the standard normal limiting distribution. When exploring the nonlinear characteristics of the time series, if the null hypothesis is rejected, it indicates that the time series has a nonlinearity.

### 2.3. Nonlinear Granger Causality Test

For the nonlinear Granger causality test, the time series needs to be filtered through the VAR model to obtain the residual series, and then the residual series is used for the nonlinear Granger causality analysis. In this study, the nonlinear Granger causality test proposed by Diks and Panchenko [27] is used.

Given there are two sets of strictly stationary time series, $X_{t}$ and $Y_{t}$, and if the past and present values of X contain the additional information about the future value of Y, then $X_{t}$ can strictly Granger cause $Y_{t}$. Let $F_{X,t}$ and $F_{Y,t}$ denote the set of past observations that contain $X_{t}$ and $Y_{t}$ before time t+1, respectively. Let ~ represent the equivalence of the distribution. The time series $X_{t}$ can strictly Granger cause $Y_{t}$ when the following conditions are met:

(6) $$(Y_{t+1},\ldots ,Y_{t+k})|\left(F_{X,t},F_{Y,t}\right)\sim (Y_{t+1},\ldots ,Y_{t+k})|F_{X,t}$$

where k≥1 represents the boundary of the predict, and at the time k=1, the conditional distribution of $Y_{t}$ is compared with and without the past and present values of $X_{t}$. Suppose the lag vector matrices $X_{t}^{L_{X}}=(X_{t-L_{X}+1},\ldots ,X_{t})$ and $Y_{t}^{L_{Y}}=(Y_{t-L_{Y}+1},\ldots ,Y_{t})$, $\left(L_{X},L_{Y}\geq 1\right)$. The null hypothesis assumes that $X_{t}^{L_{X}}$ does not contain any information that can predict the value of $Y_{t}^{L_{Y}}$, as follows:

(7) $$H_{0}:Y(t+1)|\left(X_{t}^{Lx};Y_{t}^{Ly}\right)\sim Y(t+1)|Y_{t}^{Ly}$$

For a strictly stationary bivariate time series, Equation (7) means that the distribution of the (Lx+Ly+1) dimensional vector $W_{t}=(X_{t}^{Lx},Y_{t}^{Ly},Z_{t})$ will remain constant, where $Z_{t}=Y_{t+1}$. To keep the presentation compact and easy to discuss, the time subscript is removed, and Lx=Ly=1 is assumed. Then, under these assumptions, the conditional distribution of Z, given (X,Y)=(x,y), is the same as that of Z given Y=y. Thus, Equation (7) can be re-expressed by the joint probability density function, as follows:

(8) $$\frac{f_{X,Y,Z}(x,y,z)}{f_{Y}(y)}=\frac{f_{X,Y}(x,y)}{f_{Y}(y)}\cdot \frac{f_{Y,Z}(y,z)}{f_{Y}(y)}$$

According to Equation (8), X and Z are conditional and independent of Y=y for each fixed y value, so the modified null hypothesis $H_{0}$ indicates that the following relation is established:

(9) $$q\equiv E[f_{X,Y,Z}(X,Y,Z)f_{Y}(Y)-f_{X,Y}(X,Y)f_{Y,Z}(Y,Z)]=0$$

Let ${\hat{f}}_{w}(W_{i})$ denotes the local density function estimated value of the random vector W at $W_{i}$, as follows:

(10) $${\hat{f}}_{w}(W_{i})=\frac{{\left(2\varepsilon_{n}\right)}^{-d_{W}}}{\left(n-1\right)}\sum_{j,j\neq i}I_{ij}^{W}$$

where $I_{ij}^{W}=I(\left\|W_{i}-W_{j}\right\|<\varepsilon_{n})$, I(⋅) is the index function, and $\varepsilon_{n}$ is the bandwidth parameter associated with the number of samples (n). When a local density function is given an estimation, the following test statistic is constructed:

(11) $$T_{n}(\varepsilon_{n})=\frac{n-1}{n(n-2)}\cdot \sum_{i}\left({\hat{f}}_{X,Y,Z}(X_{i},Y_{i},Z_{i}){\hat{f}}_{Y}(Y_{i})-{\hat{f}}_{X,Y}(X_{i},Y_{i}){\hat{f}}_{Y,Z}(Y_{i},Z_{i})\right)$$

For Lx=Ly=1, when $\varepsilon_{n}=Cn^{-\beta }(C>0,\frac{1}{4}<\beta <\frac{1}{3})$, the statistic $T_{n}(\varepsilon_{n})$ satisfies the following condition:

(12) $$\sqrt{n}\frac{\left(T_{n}(\varepsilon_{n})-q\right)}{S_{n}}\overset{D}{\to }N(0,1)$$

where $\overset{D}{\to }$ denotes the distribution convergence, and $S_{n}$ denotes the estimated value of the asymptotic variance of $T_{n}(\cdot )$ [27].

### 2.4. Data Sources

This study focuses on OECD countries, including Australia, Austria, Belgium, Canada, Finland, Iceland, Ireland, Japan, Korea, New Zealand, Norway, Portugal, Spain, the UK, and the US. Furthermore, five major developing countries, China as the largest developing country, India, Brazil, Mexico, and South Africa, are used for a comparative analysis with the developed countries. This study uses the annual per capita health care expenditure and per capita GDP, and the data of OECD countries ranges between 1971 and 2015. Due to the availability of the data, China’s data ranges from 1978 to 2015 and the data of the other developing countries ranges from 1995 to 2015. The per capita GDP data of the OECD and developing countries and the health expenditure care data of the four developing countries (India, Brazil, Mexico, and South Africa) is obtained from the World Bank Indicators. The health care expenditure data of the OECD is extracted from the OECD Health Database. China’s per capita health expenditure data is extracted from the 2016 Chinese Statistical Yearbook, and China’s health care expenditure per capita by the RMB exchange rate is against the dollar (the average price) and converted to USD; the RMB exchange rate against the dollar (the average price) data is also extracted from the 2016 China Statistical Yearbook. The per capita health care expenditure unit is US dollars/capita (current, US$), and the per capita GDP unit is US dollars/capita (current, US$) (It is necessary to use “constant” PPPs US dollars (Real GDP/HCE) to make a comparison between countries. However, due to the availability of the data for Health care expenditure and GDP during the period from 1971 to 2015, the data with the “current” US dollars has to be used in this study.). Table 1 is the descriptive statistics of per capita health care expenditure and per capita GDP, all of which are natural logarithms in the subsequent model calculations. Especially, the correlation coefficient for Japan is relatively lower than the ones for the other countries. It is mainly because that per capital healthcare expenditure has grown more rapidly due to that the aging population in Japan increased much more rapidly than the ones in the other countries during the sample period [38]. Therefore, it results in that the benefit growth rate of expanded health care expenditure for economic growth is relative lower.

## 3. Empirical Results

### 3.1. Unit Root Test Results

Before performing a linear Granger causality test, all the time series must be stationary, which can avoid the problem of spurious regression. Table 2 reports the results of unit root tests for all series in terms of both the per capita health care expenditure and per capita GDP using the ADF test [33]. All series in the first difference are stationary for all countries.

In addition, the structural breaks in the time series may cause the behaviour mechanism to change, thus affecting the results of the unit root test. In this study, the unit root test allows for single exogenous structural break proposed by Zivot and Andrews [39] is used. The Zivot and Andrews test is robust to a potential structural change in the time series of per capita health care expenditure in each country. As shown in Table 3, the results indicate that the first difference of the per capita health care expenditure time series in other countries is stationary except for New Zealand, Norway, and India, which are not affected by the structural breaks. Meanwhile, most of these structural breaks mainly occurred in the 1980s and 2000s. Especially, the year of break (2007/2008) for seven countries is mainly due to the exogenous shocks of the 2008 Global Financial Crisis.

### 3.2. Linear Granger Causality Results

The empirical results of the linear Granger causality between per capita health care expenditure and per capita GDP are shown in Table 4. For Ireland, Korea, Portugal, the US, China, and India, there is only a unidirectional linear Granger causality running from per capita GDP to per capita health care expenditure. A unidirectional causality running from per capita health care expenditure to per capita GDP is evident for Belgium, Norway, and Mexico. For Canada, Finland, Iceland, New Zealand, Spain, Brazil, and South Africa, there is a bidirectional causal relationship between per capita health care expenditure and per capita GDP. However, no linear causality is found for Australia, Austria, Japan, and the UK. It is worth noting that the causal test results are inconsistent with previous findings, and this may be because of different methods, data sample periods or model settings. If the relationship between health care expenditure and economic growth exhibits nonlinear characteristics, then the analysis using the linear Granger causality test model will lead to estimation bias. Therefore, this study would focus on the nonlinear causal relationship between health care expenditure and economic growth.

### 3.3. BDS Test Results

To test whether the per capita health care expenditure embeds nonlinearity characteristics, the residual time series obtained by the VAR model is performed using the BDS test. If the null hypothesis of the independent and identically distributed for the BDS test is rejected, the time series has nonlinear characteristics. Table 5 shows the BDS test results on the VAR model’s filtered residuals for the time series of per capita health care expenditure. The null hypothesis in different dimensions (m=2,3,…,10) is rejected for different countries (except for Austria, Japan, Spain, and the UK), indicating the residual series of the per capita health care expenditure is nonlinear. Therefore, the nonlinear Granger causality test is more appropriate for examining the nonlinear causal relationship between per capita health care expenditure and per capita GDP in these countries comparing to the standard linear Granger causality test.

### 3.4. Nonlinear Granger Causality Results

In this section, the nonlinear Granger causality test is implemented to deal with the residual series obtained by the VAR model to analyse the nonlinear linkage mechanism. According to Diks and Panchenko [27], the values of parameters such as the lag phase (*L*), the bandwidth parameter (*C*), the theoretical optimisation (β) and the optimal bandwidth ($\varepsilon_{n}$) should be set as $L_{x}=L_{y}=1,2,\ldots ,5$, *C* = 8, β = 2/7, and $\varepsilon_{n}$ = 1.5, respectively. Table 6 shows the results of the nonlinear relationship between per capita health care expenditure and per capita GDP, which are summarised in Table 7.

Firstly, this study reveals that for the six counties (Finland, New Zealand, Portugal, Japan, the US, and China), there is a nonlinear relationship. For Finland, New Zealand, and Portugal, a unidirectional nonlinear Granger causality exists from per capita GDP to per capita health care expenditure. For Japan, there is no linear relationship, while a unidirectional nonlinear Granger causality running from per capita health care expenditure to per capita GDP is found. For the US and China, more specifically, the nonlinear model supports a unidirectional causality from the health care expenditure to economic growth, while the linear model reveals a unidirectional causality from economic growth to health care expenditure.

Secondly, for the seven developed countries (Belgium, Canada, Iceland, Ireland, Korea, Norway, and Spain) and the four developing countries (Brazil, India, Mexico, and South Africa), there is no nonlinear Granger causality between health care expenditure and economic growth, while there exists a linear relationship between them for these countries. For Australia, Austria, and the UK, there is neither a linear Granger causality nor a nonlinear Granger causality between health care expenditure and economic growth.

In general, the nonlinear test results in this study are significantly different from those of previous studies, mainly because previous studies did not consider the structural breaks that might be related to the shocks of significant events, such as the significant changes in health policy regimes (the release of the Health Insurance Portability and Accountability Act for US in 1996) or the global financial crisis in 2008. These dramatic policy changes or financial crisis could have a different degree of impact on the causal relationship between health care expenditure and economic growth.

## 4. Discussion

Based on OECD countries and the five major developing countries (China, Brazil, India, Mexico, and South Africa), this study examines the causal relationship between health care expenditure and economic growth in these countries with considering the nonlinearity of the time series.

### 4.1. Linear Granger Causality Analysis

The linear Granger causality results for Ireland, Korea, Portugal, the US, China, and India indicate that economic growth has led to an increase in health care expenditure. In other words, the GDP growth in these six countries may mainly rely on scientific and technological development or infrastructure investment, such as information and communication technology, and roughly 9% of its GDP invested in infrastructure for China in the 1990s and 2000s. Therefore, the effect of the related policies on health care expenditure for the promotion of GDP is limited. However, the results for Belgium, Norway, and Mexico show that there exists a unidirectional causality running from per capita health care expenditure to per capita GDP. Therefore, the role of health care expenditure in these three countries is equivalent to the positive effect of education, which has become one impetus to promote the national economy’s growth. The results for Canada, Finland, Iceland, New Zealand, Spain, Brazil, and South Africa show that there is a bidirectional linear causality between per capita health care expenditure and per capita GDP. In these countries, economic growth and health expenditure mutually affect each other. However, the results for Australia, Austria, Japan, and the UK show that there is no linear causality between per capita health care expenditure and per capita GDP; here, the health care expenditure also has no linear effect on economic growth.

### 4.2. Nonlinear Granger Causality Analysis

The results of the nonlinear test of Finland, New Zealand, Japan, the US, and China are not consistent with the results of the linear test. However, the results of the nonlinear test of Portugal are consistent with the results of the linear test. For Finland and New Zealand, there is a unidirectional nonlinear Granger causality running from per capita GDP to per capita health care expenditure, whereas a bidirectional linear Granger causality is found for both countries (The results interestingly found that there exists both linear and nonlinear causality from GDP to health care expenditure in Finland and New Zealand. The causality can be linear and nonlinear, which is not contradictory, because that the linear causality can only be meaningfully defined for systems with linear interactions among their variables, but the nonlinear causality may exist in the nonlinear systems. The systems for the health expenditure and GDP in Finland and New Zealand may have linear and nonlinear characteristics.). There exists a unidirectional nonlinear Granger causality running from per capita health care expenditure to per capita GDP for Japan, while there is no linear relationship. A unidirectional linear causality from economic growth to health care expenditure is found for the US and China, while there exists a unidirectional nonlinear causality from the health care expenditure to economic growth for them. The reason for these changes may be due to the fact that linear Granger causality model cannot account for non-linearities caused by the structural breaks, which may be related to significant economic events. It has been shown that the time series for the health care expenditure or GDP would been influenced by significant economic events. The structural breaks are identified most often around recessions, such as the global financial crisis in 2008. Some reforms related to the health systems, such as promoting and extending the health coverage and benefits and the reimbursement policies of health care expenditure may cause the structural changes of time series for the health care expenditure. Other significant changes of institutional factors related health care (such as the regulations on health care insurance markets, and the dramatic innovations of medical procedures and technology) can also cause some dramatic changes in health care expenditure. Furthermore, some extreme events may make the changes of the economic relationships—for instance, the economic recessions caused by the global financial crisis may make health policymakers adopt related important measures to control the health care expenditure. Then, when exploring the causal relationship between health care expenditure and economic growth, the non-linearities have to be addressed. Therefore, when the nonlinearity has been considered in the nonlinear Granger causality test, different conclusions would be obtained compared to the results of the linear Granger causality test.

For other countries, no nonlinear causal relationship is found for them. However, some considerable differences exist in the results for them. Some of them (seven developed countries: Belgium, Canada, Iceland, Ireland, Korea, Norway, and Spain and four developing countries: Brazil, India, Mexico, and South Africa) have linear causal relationship between the health care expenditure and economic growth. The main reason may be that that the nonlinear relationship is unable to clearly point out the casual relationship. On the contrary, neither a linear Granger causality nor a nonlinear Granger causality between health care expenditure and economic growth can be found for Australia, Austria, and the UK. The main reason for Australia and the UK is that these two countries have primarily tax financed health systems, so in theory GDP could have a bearing on tax revenues, which could fund health care expenditure. However, the main reason for Austria is due to the public share of health care expenditures has been usually substantially high, and Austria usually has better public infrastructure and rich medical and health care resources, which could lower the significance of per capita GDP in explaining the per capita health care expenditure. Furthermore, the positive externality of the health care expenditures may reduce the influence of the economic growth on the health care expenditure. Meanwhile, the economic growth of these three developed countries mainly depend on technology change, such as the reorganization of economies mainly caused by the productivity and mechanization obtained from the technology change, which decrease the dependence of the economic growth on the health care expenditure.

### 4.3. Limitations and Strength

Although some interesting findings are obtained in this study, several limitations should be addressed. Firstly, the time span of the major developing countries is relative short due to the availability of the data. This may have impact on the robustness of the causal results for the major developing countries. Secondly, the characteristics that some of the structural breaks are detected for the health care expenditure and GDP are mainly due to the policies and extreme economic events, which indicates that some instability may be exhibited when modelling the complex causal relationship. The future research would investigate these concerns.

## 5. Conclusions and Policy Implications

The causal relationship between health care expenditure and economic growth has always been a theme worth studying. Health care expenditure is significant for any country’s economic development and hence plays a very important role in the economic activity, the society’s security, and the social welfare. As shown numerous times before, a higher level of economic development can lead to greater health spending. For the improvement of national health and quality of life, more and more countries have developed health policies to regulate the health care expenditure and enhance the efficiency of the health spending. Therefore, the impact of the corresponding changes in health policy and related fiscal policy of the health spending on economic growth has received more and more attention.

Different from previous studies, the linear Granger causality test, in addition to the nonlinear Granger causality test, are applied to explore the complex causality between health care expenditure and economic growth for 15 OECD countries and 5 major developing countries. The results show that there is a nonlinear Granger causality relationship between health care expenditure and economic growth in six countries: Finland, Japan, New Zealand, Portugal, the US, and China. The existence of this nonlinearity is mainly due to structural breaks caused by some significant events such as the significant changes in health policy regimes (the release of the State Children’s Health Insurance Program and the Benefits Improvement and Protection Act for US in 2000, and the introduction of the New Rural Co-operative Medical Care Scheme for China in 2002) or the economic recession in some country or the world, but the linear model cannot completely capture the nonlinear information.

For Australia, Austria, and the UK, there is neither a linear Granger causality nor a nonlinear Granger causality between health care expenditure and economic growth. However, a unidirectional linear causal relationship running from economic growth to health care expenditure exists for Ireland, Korea, Portugal, and India. For these six countries, the health care expenditure cannot effectively enhance economic growth, which may be due to the different levels of economic growth. Thus, the role of health care expenditure for the economic growth also changes. According to implications that could have on policy, the implementation of related policy and strategies on health care expenditure would not significantly impact the economic growth of these countries. However, the improvements in the health systems would be still worth the effort for the improvement of the health outcomes, such as the improvement of the life expectancy and the life quality of the population. Therefore, these countries need to make the health spending more effective in improving the health outcomes.

On the other hand, a unidirectional linear causality running from health expenditure to economic growth is found for Belgium, Norway, and Mexico. Moreover, a bidirectional linear causality exists for Canada, Finland, Iceland, New Zealand, Spain, Brazil, and South Africa. Especially for the US, China, and Japan, there is a unidirectional nonlinear causal relationship running from health expenditure to economic growth. For these countries, an increase in health care expenditure can effectively enhance the economic growth. It means that the efficient and reliable healthcare system is able to construct a high-quality economic growth. Therefore, the implementation of fiscal policy related to health care expenditure would have a positive impact on economic growth. To improve the quality of national health, life quality, and happiness, the above countries need to increase the investment in the research of the medical technologies in order to make more effective treatment for patients, and also increase the investment in the other health care aspects to improve the quality of health systems. Furthermore, they should actively look for optimising policy related to health care expenditure, such as by standardising the detail statement of the health care expenditure, improving the efficiency of health spending and so on to promote the rapid and sustainable economic development.

## Figures and Tables

**Table 1:** Summary statistics for per capita health care expenditure and per capita Gross Domestic Product (GDP).

Country	Var.	Mean	Median	Max.	Min.	Std. Dev.	Corr.
Australia	HCE	1764.539	1381.114	4420.436	223.592	1265.098	0.955
	GDP	23,750.480	18,591.220	67,652.680	3487.615	18,157.970	
Austria	HCE	2096.557	1874.612	5015.637	201.962	1506.272	0.975
	GDP	24,235.000	24,489.740	51,386.380	2375.243	15,781.410	
Belgium	HCE	1897.080	1611.027	4611.252	168.506	1355.471	0.967
	GDP	23,307.980	23,121.570	48,424.590	3099.433	14,318.330	
Canada	HCE	2106.189	1966.955	4608.452	324.051	1352.123	0.980
	GDP	23,628.860	20,771.250	52,496.690	4586.256	14,514.540	
Finland	HCE	1635.753	1418.182	3983.543	190.192	1173.899	0.970
	GDP	24,502.700	24,253.250	53,401.310	2718.208	15,398.370	
Iceland	HCE	1973.016	1747.649	4011.997	227.884	1218.395	0.945
	GDP	27,707.840	26,851.020	68,348.320	3278.649	16,734.760	
Ireland	HCE	1729.788	980.492	5130.683	149.630	1630.996	0.960
	GDP	23,685.650	15,729.930	61,388.170	1705.618	20,505.250	
Japan	HCE	1618.011	1371.314	4152.373	157.096	1200.219	0.841
	GDP	25,747.620	31,902.770	48,629.200	2234.262	14,666.860	
Korea	HCE	705.887	387.344	2487.939	16.036	759.137	0.970
	GDP	10,123.460	8140.219	27,989.350	316.831	8722.995	
New Zealand	HCE	1414.995	1105.321	3590.169	214.800	1026.355	0.977
	GDP	16,889.470	13,663.200	44,380.430	2773.323	11,833.470	
Norway	HCE	2307.469	1574.586	6567.032	156.778	1979.912	0.972
	GDP	39,417.420	29,315.840	10,2910.400	3736.349	30,949.150	
Portugal	HCE	1112.026	808.512	2645.663	54.669	911.802	0.981
	GDP	10,482.880	9978.302	24,815.610	1064.643	7751.727	
Spain	HCE	1280.525	1048.008	3152.987	109.724	1007.855	0.972
	GDP	14,817.410	14,676.710	35,578.740	1358.466	10,436.130	
UK	HCE	1490.169	1144.029	4003.002	162.026	1162.423	0.959
	GDP	22,427.540	19,900.730	49,949.150	2649.802	14,940.980	
US	HCE	3800.523	3299.976	9451.342	357.983	2826.105	0.991
	GDP	28,362.200	26,464.850	56,115.720	5623.444	15,662.080	
China	HCE	88.790	28.910	478.582	6.800	126.931	0.996
	GDP	1781.160	745.579	8069.212	156.396	2322.355	
Brazil	HCE	540.424	391.294	1055.136	201.094	305.7375	0.994
	GDP	6912.914	5271.411	13,167.47	2819.65	3523.996	
India	HCE	32.49,392	27.75,145	63.31,774	15.822	15.39,868	0.989
	GDP	857.811	707.008	1596.47	370.1014	456.468	
Mexico	HCE	425.2072	472.1081	617.8747	172.44	145.9076	0.985
	GDP	7640.788	7748.123	10,452.78	3655.598	2060.015	
South Africa	HCE	367.5331	354.0624	597.3594	169.0222	127.3705	0.965
	GDP	4918.76	5277.925	7976.466	2461.355	1736.789	

Notes: HCE represents health care expenditure. Var. = variable; Max. = maximum; Min. = minimum; Std. Dev. = standard deviation; Corr. indicates the correlation coefficient between HCE and GDP in each country.

**Table 2:** The results of augmented Dickey-Fuller (ADF) unit root tests for per capita health care expenditure and per capita GDP.

Country	HCE		GDP	
	Level	First Difference	Level	First Difference
Australia	−2.912(0)	−5.579(0) ***	−3.388(1) *	−4.739(0) ***
Austria	−2.194(0)	−5.256(0) ***	−2.285(0)	−4.646(0) ***
Belgium	−4.616(1) ***	−4.287(0) ***	−2.836(1)	−4.074(0) **
Canada	−1.879(1)	−3.768(0) **	−2.365(1)	−4.258(0) ***
Finland	−2.010(1)	−4.139(0) **	−2.874(1)	−4.304(0) ***
Iceland	−1.912(1)	−5.424(0) ***	−3.324(1) *	−4.727(0) ***
Ireland	−2.430(1)	−3.768(0) **	−1.959(1)	−5.029(0) ***
Japan	−2.643(1)	−4.241(0) ***	−1.166(1)	−4.970(0) ***
Korea	−0.964(0)	−6.279(0) ***	−1.644(0)	−5.760(0) ***
New Zealand	−3.703(0) **	−5.779(0) ***	−4.124(1) **	−4.261(0) ***
Norway	−2.202(0)	−6.791(0) ***	−2.475(1)	−4.045(0) **
Portugal	−2.621(0)	−6.106(0) ***	−1.703(1)	−4.285(0) ***
Spain	−1.604(1)	−4.568(0) ***	−2.337(1)	−4.047(0) **
UK	−2.288(0)	−5.776(0) ***	−0.530(6)	−5.406(4) ***
US	−2.305(1)	−3.434(1) *	−1.894(0)	−4.916(0) ***
China	−1.004(0)	−5.845(1) ***	−2.130(1)	−4.009(0) **
Brazil	0.281(1)	−2.386(0) **	0.692(0)	−2.541(0) **
India	−2.206(0)	−4.852(0) ***	−3.062(4)	−3.444(0) *
Mexico	−0.289(0)	−5.538(0) ***	−1.939(0)	−4.145(4) **
South Africa	0.575(0)	−2.852(0) ***	0.674(0)	−2.659(0) **

Notes: HCE represents per capita health care expenditure. The regressions include an intercept and trend. All variables are in natural logarithms while the optimal lag length is determined via the SIC criterion and are in parentheses. The numbers in parentheses are the optimal lag order. *, **, or *** denote that the null hypothesis is rejected at the 10%, 5%, or 1% significance levels, respectively.

**Table 3:** Zivot–Andrews unit root test (with one structural break) for per capita health care expenditure.

Country	Level		First Difference	
	Test Value	Year of Break	Test Value	Year of Break
Australia	−4.648(1)	2008	−7.223(1) ***	1982
Austria	−3.079(0) ***	1990	−6.463(0) **	1985
Belgium	−4.047(0) *	2008	−6.645(0) *	1983
Canada	−3.127(1) *	1980	−4.590(0) ***	1997
Finland	−3.490(4)	1986	−3.334(3) *	1992
Iceland	−3.004(2) **	2003	−5.849(0) *	1989
Ireland	−2.499(1)	2008	−4.828(0) **	2000
Japan	−5.147(1) *	1983	−5.033(0) **	1989
Korea	−4.624(4)	1990	−6.585(0) **	1982
New Zealand	−6.280(2) **	2008	−6.709(0)	1981
Norway	−3.449(0) *	2003	−7.216(0)	1983
Portugal	−3.139(4)	2008	−8.703(3) **	1999
Spain	−3.101(1)	2007	−5.650(0) **	1987
UK	−2.997(0)	2007	−3.819(5) *	2000
US	−2.996(2)	1988	−4.872(1) ***	2000
China	−2.943(2)	2000	−6.568(1) **	1988
Brazil	−3.192(3)	2012	−4.653(0) **	2002
India	−3.510(0) **	2004	−5.779(0)	2002
Mexico	−2.726(0)	2005	−5.146(4) *	2010
South Africa	−3.210(1)	2000	−5.912(0) ***	2001

Notes: *, **, or *** denote that the null hypothesis is rejected at the 10%, 5%, or 1% significance levels, respectively. The number in parentheses is the optimal lag order. The lag parameters are selected based on the AIC.

**Table 4:** Results of the linear Granger causality between per capita health care expenditure and per capita GDP.

Country	Lag	*H*_0_: HCE Does Not Cause GDP		*H*_0_: GDP Does Not Cause HCE		Results
		$\chi^{2}\;$	*p*-Value	$\chi^{2}\;$	*p*-Value	
Australia	1	1.997	0.1576	0.536	0.4641	×
Austria	1	1.598	0.2062	1.983	0.1590	×
Belgium	2	5.880	0.0529	2.932	0.2309	HE=>GDP
Canada	2	8.308	0.0157	7.725	0.0210	HE<=>GDP
Finland	2	12.625	0.0018	10.377	0.0056	HE<=>GDP
Iceland	2	14.420	0.0007	5.806	0.0549	HE<=>GDP
Ireland	1	0.001	0.9766	8.553	0.0035	HE<=GDP
Japan	1	0.054	0.8156	0.327	0.5673	×
Korea	1	0.796	0.3724	5.783	0.0162	HE<=GDP
New Zealand	2	16.625	0.0002	11.370	0.0034	HE<=>GDP
Norway	1	3.561	0.0592	0.203	0.6523	HE=>GDP
Portugal	1	0.262	0.6088	3.071	0.0797	HE<=GDP
Spain	2	8.101	0.0174	12.706	0.0017	HE<=>GDP
UK	1	1.128	0.2881	1.645	0.1996	×
US	2	1.889	0.3889	7.463	0.0240	HE<=GDP
China	1	0.691	0.4060	5.887	0.0153	HE<=GDP
Brazil	1	8.552	0.0035	6.823	0.0090	HE<=>GDP
India	1	2.585	0.1079	3.053	0.0806	HE<=GDP
Mexico	1	3.060	0.0802	0.4520	0.5014	HE=>GDP
South Africa	1	8.169	0.0043	7.037	0.0080	HE<=>GDP

Note: The optimal lag order is determined based on the SIC criterion. HCE represents per capita health care expenditure.

**Table 5:** Results of BDS test from the Vector autoregression (VAR) model filtered residuals of the per capita health care expenditure.

Embedding Dimension (*m*)	BDS Statistics for Countries									
	Australia	Austria	Belgium	Canada	Finland	Iceland	Ireland	Japan	Korea	New Zealand
2	0.033 **	0.010	0.007	0.010	0.005	0.035 **	0.031 ***	0.007	0.066 ***	0.030 ***
3	0.053 **	0.026	0.004	0.011	−0.005	0.043 *	0.041 ***	0.021	0.110 ***	0.057 ***
4	0.045 *	0.018	0.013	0.036 **	0.029	0.040	0.057 ***	0.027	0.132 ***	0.075 ***
5	0.061 **	0.007	0.027	0.052 ***	0.044 *	0.041	0.078 ***	0.034	0.159 ***	0.078 ***
6	0.076 ***	0.002	0.018	0.037 **	0.045 *	0.043	0.086 ***	0.006	0.164 ***	0.079 ***
7	0.086 ***	0.015	0.033	0.052 ***	0.042 *	0.041	0.087 ***	0.017	0.146 ***	0.081 ***
8	0.086 ***	0.020	0.039 *	0.041 ***	0.030	0.034	0.077 ***	0.020	0.128 ***	0.076 ***
9	0.095 ***	0.020	0.039 **	0.041 ***	0.018	0.024	0.068 ***	0.015	0.106 ***	0.067 ***
10	0.097 ***	0.017	0.047 ***	0.032 ***	0.010	0.016	0.057 ***	0.013	0.082 ***	0.052 ***
	**Norway**	**Portugal**	**Spain**	**UK**	**US**	**China**	**Brazil**	**India**	**Mexico**	**South Africa**
2	0.012	0.026 **	−0.011	−0.018	−0.013	0.060 ***	0.139 ***	0.167 **	0.207 **	0.113 **
3	0.010	0.040 **	0.002	−0.035	−0.034*	0.095 ***	0.207 **	0.267 **	0.353 **	0.178 **
4	0.012	0.071 ***	0.018	−0.042	−0.014	0.110 ***	0.210 **	0.315 **	0.454 **	0.190 **
5	0.022	0.084 ***	0.013	−0.004	0.005	0.113 ***	0.176 **	0.317 **	0.525 **	0.148 **
6	0.038	0.093 ***	0.019	0.013	0.014	0.106 ***	0.094 **	0.250 **	0.574 **	0.063 **
7	0.048	0.101 ***	0.010	−0.009	0.035*	0.101 ***	−0.009 **	0.080 **	0.609 **	−0.045 **
8	0.057 **	0.104 ***	0.006	0.008	0.047**	0.100 ***	−0.121 **	0.071 **	0.632 **	−0.070 **
9	0.056 **	0.102 ***	0.001	0.019	0.042**	0.094 ***	−0.368 **	0.016 **	0.654 **	−0.060 **
10	0.053 ***	0.092 ***	−0.002	0.024	0.045***	0.092 ***	−0.545 **	−0.152 **	0.676 **	−0.063 **

Note: *, **, or *** indicate significant nonlinear dependencies at the 10%, 5%, or 1% levels, respectively.

**Table 6:** Results of the nonlinear Granger causality exploration between per capita health care expenditure and per capita GDP.

*L_x_ = L_y_*	*H*_0_: HCE Does Not Cause GDP	*H*_0_: GDP Does Not Cause HCE	*H*_0_: HCE Does Not Cause GDP	*H*_0_: GDP Does Not Cause HCE
	*T_n_*	*T_n_*	*T_n_*	*T_n_*
	Australia		Korea	
1	−0.200	0.378	0.862	−0.961
2	−0.851	0.526	0.328	0.813
3	0.065	0.036	−0.160	1.181
4	−0.045	0.103	0.676	0.885
5	−0.646	1.075	0.214	0.505
	Austria		New Zealand	
1	−1.057	1.075	−1.239	1.551 *
2	−1.626	−1.201	−1.684	0.767
3	−1.139	−0.232	−1.121	0.949
4	−1.160	0.049	−1.011	0.498
5	−0.523	1.035	−0.735	−0.341
	Belgium		Norway	
1	−2.349	−1.411	−1.183	−2.147
2	−1.252	−0.686	−1.011	−1.904
3	−0.836	0.062	−0.581	−1.138
4	−0.164	0.572	0.452	−0.592
5	−0.623	1.137	0.635	−0.543
	Canada		Portugal	
1	1.126	0.492	−0.830	−0.342
2	0.808	−0.015	0.948	1.409 *
3	0.896	−0.247	0.998	1.093
4	0.604	−0.364	0.835	0.878
5	1.037	−0.000	1.155	0.390
	Finland		Spain	
1	0.286	1.842 **	−0.084	0.099
2	0.807	1.293 *	−0.252	0.186
3	0.864	1.590 *	−0.174	−0.776
4	0.263	1.422 *	0.771	0.165
5	0.093	1.082	0.347	1.011
	Iceland		UK	
1	0.279	−0.547	−1.171	0.525
2	0.786	−0.513	−0.624	0.397
3	1.094	−0.564	−0.360	0.716
4	0.275	−0.789	−0.292	1.047
5	−0.000	−1.037	0.419	1.102
	Ireland		US	
1	0.279	−0.547	−0.907	−0.778
2	0.786	−0.513	−0.602	−0.858
3	1.094	−0.564	0.276	0.051
4	0.275	−0.789	1.345 *	0.314
5	−0.000	−1.037	1.244	−0.361
	Japan		China	
1	1.285 *	0.238	1.157	0.614
2	0.995	−0.187	1.533 *	0.987
3	1.270	−1.294	0.969	0.451
4	1.357 *	−1.066	1.226	0.399
5	1.280	−0.940	0.932	0.721
	Brazil		India	
1	−0.877	−0.687	−0.326	−1.152
2	−0.849	−0.696	−0.394	−1.182
3	−0.871	−0.707	1.228	−1.231
4	−0.874	−0.577	1.234	−1.193
5	−0.736	−0.191	1.246	−1.141
	Mexico		South Africa	
1	−0.687	0.627	−0.757	−0.802
2	−0.786	0.310	−0.771	−0.819
3	−0.802	0.336	−0.789	−0.839
4	−0.802	0.409	−0.792	−0.850
5	−0.736	0.542	−0.752	−0.804

Note: *, **, or *** denote that the null hypothesis is rejected at the 10%, 5%, or 1% significance levels, respectively. HCE represents per capita health care expenditure.

**Table 7:** Linear and nonlinear Granger causality between per capita health care expenditure and per capita GDP.

Country	Linear Granger Causality	Nonlinear Granger Causality
Australia	×	×
Austria	×	×
Belgium	HCE =>GDP	×
Canada	HCE<=>GDP	×
Finland	HCE<=>GDP	HCE<=GDP
Iceland	HCE<=>GDP	×
Ireland	HCE<=GDP	×
Japan	×	HCE=>GDP
Korea	HCE<=GDP	×
New Zealand	HCE<=>GDP	HCE<=GDP
Norway	HCE=>GDP	×
Portugal	HCE<=GDP	HCE<=GDP
Spain	HCE<=>GDP	×
UK	×	×
US	HCE<=GDP	HCE=>GDP
China	HCE<=GDP	HCE=>GDP
Brazil	HE<=>GDP	×
India	HE<=GDP	×
Mexico	HE=>GDP	×
South Africa	HE<=>GDP	×

Note: HCE represents per capita health care expenditure.

## Supplementary Materials

The following are available online at http://www.mdpi.com/1660-4601/15/9/1953/s1, Table S1: Per capital GDP, Table S2: Per capital Health expenditure.
